# Comparison of prognosis of patients with endometrial cancer after hysteroscopy versus dilatation and curettage: A multicenter retrospective study

**DOI:** 10.3389/fmed.2022.1097133

**Published:** 2023-01-09

**Authors:** Shihuang Liu, Lan Zhen, Shaoyu Zhang, Yurong Cai, Yanying Lin, Fulian Chen, Xiaowen Li, Qianru You, Xiaohong Lai, Hangbo Lai, Xiangqin Zheng, Huan Yi

**Affiliations:** ^1^Department of Gynecology Oncology, Fujian Provincial Maternity and Children’s Hospital, Fujian Provincial Key Gynecology Clinical Specialty, The Affiliated Hospital of Fujian Medical University, Zhangzhou, Fujian, China; ^2^Department of Gynecology, Fuding Municipal Hospital, Zhangzhou, Fujian, China; ^3^Department of Gynecology, Zhangpu County Traditional Chinese Medicine Hospital, Zhangzhou, Fujian, China; ^4^Department of Gynecology, Zhangzhou Hospital Affiliated to Fujian Medical University, Zhangzhou, Fujian, China

**Keywords:** endometrial cancer, hysteroscopy, peritoneal cytology, prognosis, curettage

## Abstract

**Introduction:**

Hysteroscopy is a useful procedure for diagnosing endometrial cancer. There is controversy regarding whether hysteroscopy affects the prognosis of endometrial cancer by prompting cancer cell into intraperitoneal dissemination. Our purpose was to confirm whether hysteroscopy could be a risk factor of the tumor stage, recurrence and survival rate of endometrial cancer.

**Methods:**

This multicenter retrospective study included all consecutive patients who had endometrial carcinoma diagnosed preoperatively with hysteroscopy and directed endometrial biopsy (HSC, group A) and dilatation and curettage (D&C, group B) between February 2014 and December 2018 at the Fujian Provincial, China. We compared the demographic feature, clinical characteristics and prognosis between the two groups.

**Results:**

A total of 429 patients were included in the study (Group A, *n* = 77; Group B, *n* = 352). There was no significant difference between their baseline characteristics [including age, BMI, histological type and International Federation of Gynecology and Obstetrics (FIGO) stage]. By comparing several pathological conditions that may affect prognosis, there were no significant differences between the two groups in the peritoneal cytology, depth of myometrial invasion, the positivity of lymph nodes, lymphovascular space invasion and paraaortic lymph node dissection. Finally, no significant difference was found between the two groups in overall survival (OS) (*P* = 0.189) or recurrence free survival (RFS) (*P* = 0.787).

**Conclusion:**

Under certain inflation pressure and distension medium, hysteroscopic examination and lesion biopsy ensure the safety and have no adverse effects on prognosis compared to conventional curettage.

## Introduction

Endometrial cancer, a tumor originating in the uterine endometrium, is one of the most common gynecological cancers, with its prevalence has increased worldwide in recent years (1). As the disease is frequently symptomatic at an early stage in the majority of patients, endometrial cancer is often diagnosed at stage I, which means the disease is confined to the uterus. The 5-year survival rates are as high as 74–91% in these stage I patients (1, 2). Therefore, it is important to diagnose endometrial cancer at an early stage. The current diagnosis of endometrial cancer is based on histological results of endometrial sampling by endometrial biopsy, uterine dilation and curettage (D&C), and hysteroscopy and directed endometrial biopsy (HSC). D&C is a common diagnostic procedure. The tools of D&C are more readily available, and the procedures are more mature and easily quality-controlled, thus facilitating the implementation of D&C in a wide range of primary care hospitals in China. Although D&C is a common diagnostic blind procedure for endometrial cancer in all institutions (3), it might lead to a high false negative rate in endometrial cancer (4). In contrast, visible HSC has increasing used to determine endometrial lesions, especially in minimal lesions, and performing biopsies. Considered HSC provides direct visualization of the endometrial cavity, hysteroscopy-guided biopsy has a high accuracy for the diagnosis of endometrial cancer (5).

Recently, hysteroscopy is considered to be a standard procedure for diagnosing endometrial cancer (4, 6–8). Especially, for young women who wants to preserve fertility, hysteroscopy can preserve the integrity of the endometrium to the greatest extent, offering the possibility of fertility for patients with early stage endometrial cancer and reducing the incidence of adverse pregnancies and deliveries (9, 10) and for postmenopausal women, the hysteroscopic visual appearance could detect morphological differences in endometrial cancer (11, 12). Hysteroscopy can also underly the advantages of performing hysteroscopy in the preoperative management of endometrial cancer, as it allows the distinction between endocervical mucosal infiltration and a protrusion into the endocervical canal, helping to inform the decision on therapeutic management. However, some studies have shown that hysteroscopy has the potential risk to cause the spread of cancerous cell (13) and manifest side effects in endometrial prognosis. Therefore, we conducted this retrospective study to compare the risk factors, recurrence and survival rate of women with endometrial cancer between HSC and D&C as the diagnostic procedure.

## Patients and methods

### Patients

This multicenter retrospective study included all consecutive patients who had endometrial carcinoma diagnosed preoperatively with either D&C or HSC between February 2014 and December 2018 at the Fujian Provincial, China. Patient stage of endometrial cancer was based on the International Federation of Gynecology and Obstetrics (FIGO) 2009 staging system. The patients were divided into two groups by the diagnostic procedure: HSC (Group A) versus D&C (Group B).

Women were excluded as follows: (1) had not undergone hysterectomy and bilateral salpingo-oophorectomy (HBSO) with washing for cytology; (2) had not undergone neither D&C or HSC before HBSO; (3) incomplete follow-up. The study was approved by our institution’s ethics committee (2022KYLLR0343).

The clinical, surgical, and pathological results were retrieved from electronic dataset for analysis. A detailed analysis of tumor histopathological risk characteristics was performed, including histopathological type, tumor differentiation, the depth of myometrial invasion, lymph-vascular invasion and FIGO stage. Follow-up information were recorded by outpatient department and telephone. Our cohort of patients was followed every 3 months from the date of surgery until an event (recurrence, death from disease, or death) or the last follow-up up to December 2020.

### Surgical procedure

HSC was performed under general anesthesia. A saline solution warmed to body temperature was used as the distension medium. In the procedure, the distension medium was installed into the pressure cuff, and the intrauterine pressure was set between 20 and 23 kPa. Intrauterine pressure was controlled with a Vario Flow device (Olympus). We used hysteroscopy to examine the cervical canal, anterior and posterior walls, both sides of the wall, the fundus of the uterine cavity and suspicious lesions, all of which were sampled at multiple points. The sample technique is using a hysteroscopic 5Fr toothed grasping forceps to “plow” along with the suspicious tissue for about 0.5–1 cm. And then grasping forceps retrieved from the uterine cavity together along with the hysteroscope, without, retracting the tip of the forceps into the operating channel. D&C was also performed under general anesthesia. Curettage of the cervical canal and the uterine cavity was performed separately. Tissue samples for histological examination were obtained during both procedures.

During the comprehensive staging surgery for endometrial carcinoma, samples of peritoneal washings with saline solution was performed to obtain cytological examination in cases with no free fluid. The samples were inspected by an expert cytopathologist. In cases of small numbers of positive cells after immunostaining, peritoneal cytology was described as suspicious. We therefore included suspicious results in the analysis of positive peritoneal cytology.

### Statistical analysis

Statistical analysis was performed with SPSS software version 22.0 (IBM, Armonk, NY, USA). Descriptive analysis, chi-square tests and t tests of independent samples were performed as applicable. A *p*-value of less than 0.05 was considered statistically significant. We conducted a series of survival analyses using Kaplan-Meier statistics. The significance of the difference in the unadjusted survival curves was assessed using the log-rank test.

## Results

### Baseline characteristics

**FIGURE 1:**
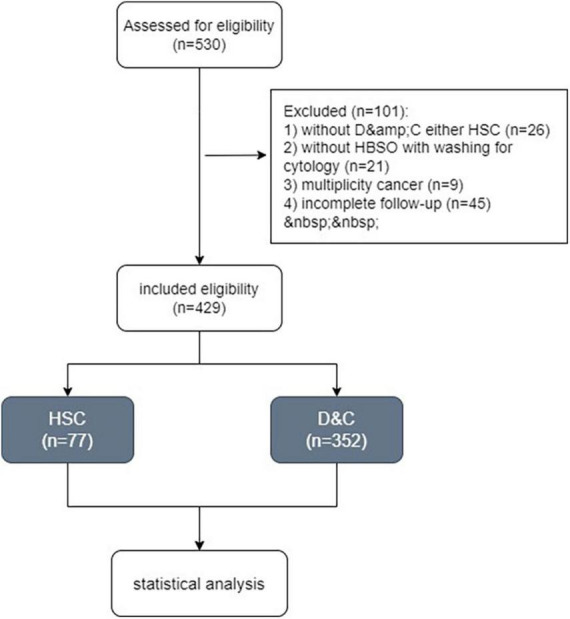
Patients collection flowchart.

Totally, 530 women with endometrial carcinoma were recorded between February 2013 and December 2018. Finally, 429 patients who met our criteria underwent HSC (Group A, *n* = 77) and D&C (Group B, *n* = 352) were included in the study (Figure 1).

The baseline characteristics of the two groups are presented in Table 1. The mean age was 52.36 years in Group A and 53.99 years in Group B, and the mean BMI was 24.74 kg/m^2^ in Group A and 24.79 kg/m^2^ in Group B. There was no significant difference between the two groups. In terms of postoperative pathology, endometrioid adenocarcinoma was predominant in both groups (*N* = 371; 86.5%). Among the endometrioid adenocarcinomas, differentiation was predominantly G1 and G2, and there was no significant difference between the two groups. According to 2009 FIGO staging system, early stage was predominant in both groups, and there was no significant difference.

**TABLE 1:** Baseline characteristics of HSC and D&C.

Baseline characteristics	Hysteroscopy *n* = 77 (%)	D&C *n* = 352 (%)	*P-*value
Age (years, mean ± S/D)	52.36 ± 7.51	53.99 ± 8.35	0.115
BMI (kg/m^2^, mean ± S/D)	24.74 ± 3.52	24.79 ± 3.59	0.911
Histological type, *n* (%)			0.518
Endometroid adenocarcinoma	70 (90.9)	320 (90.9)	
Non-endometroid adenocarcinoma	7 (9.1)	32 (9.2)	
Histological grade			0.809
G1	34 (54.8)	172 (57)	
G2	22 (35.5)	108 (35.8)	
G3	6 (9.7)	22 (7.3)	
FIGO stage			0.435
Early stage (I–II)	71 (92.2)	321 (91.2)	
Advanced stage (III–IV)	6 (7.8)	29 (8.3)	

### HSC impact on prognostic high-risk factors for endometrial cancer

As mentioned in many studies, HSC can lead to positive peritoneal cytology; however, the correlation between positive peritoneal cytology and tumor progression is more controversial. And HSC may cause poor prognosis by myometrial invasion, lymph node metastasis, and lymph-vascular invasion for the inflation pressure (14, 15). Therefore, by comparing several results from HSC pathological risk conditions that may affect prognosis (13, 16–18), such as the depth of myometrial invasion, lymph node metastasis, and lymph-vascular invasion, there was no significant difference between the 2 groups (Table 2).

**TABLE 2:** Comparing pathological conditions that may affect the prognosis of EC between HSC and D&C.

High-risk factors	Hysteroscopy *n* = 77 (%)	D&C *n* = 352 (%)	*P*-value
**Myometrial invasion**			0.191
None	11 (14.3)	41 (11.8)	
Less than 1/2	44 (57.1)	237 (68.1)	
More than 1/2	22 (28.6)	70 (20.2)	
Positivity of lymph nodes	5 (6.5)	18 (5.1)	0.398
Pelvic lymph node	2 (2.6)	14 (4.0)	0.429
Para-aortic lymph node	3 (3.9)	4 (3.9)	0.113
Lymph node dissection			
Pelvic lymph node dissection	22 (28.6)	163 (46.3)	0.005
Para-aortic lymph node dissection	12 (15.6)	40 (11.4)	0.525
Lympho-vascular space invasion			0.459
Present	6 (7.8)	24 (6.8)	
Absent	71 (92.2)	328 (93.2)	
Peritoneal cytology			0.442
Positive or suspicious	3 (4.3)	9 (2.7)	
Negative	67 (95.7)	327 (97.3)	

### Analysis of cases of positive peritoneal cytology

Peritoneal dissemination is most likely to be influenced by HSC (7, 8, 15, 19). However, in our study, there was no significant difference between the two groups. Therefore, we would like to further explore the factors associated with peritoneal dissemination. Only a total of 12 patients in the two groups showed positive or suspicious cytology in the peritoneal washings, and the detailed cases of the results are shown in Table 3.

**TABLE 3:** Description of 12 patients with positive peritoneal cytology.

Study ID	Age	Method of diagnosis	Diagnosis	FIGO stage	Myometrial invasion	LVSI	Distant metastasis
54	61	D&C	Endometrioid carcinoma 70% mixed serous carcinoma 30%	IIIc1	>1/2	Y	Pelvic lymph node
63	61	D&C	Endometrioid carcinoma FIGO G2	IIIA	>1/2	N	Bilateral accessary
139	61	Hysteroscopy	Non-keratinized squamous cell carcinoma	IIIC2	>1/2	Y	Para-aortic lymph node
158	46	D&C	Endometrioid carcinoma FIGO G1	IA	<1/2	N	None
174	50	D&C	Endometrioid carcinoma FIGO G1	II	<1/2	N	Cervix
211	43	D&C	Endometrioid carcinoma FIGO G3 mixed clear cell carcinoma	IIIC1	>1/2	Y	Pelvic lymph node
240	41	D&C	Endometrioid carcinoma FIGO G1	IA	>1/2	N	None
243	61	D&C	Serous carcinoma	IVB	>1/2	Y	Peritoneal, Omental, and accessary (L)
289	52	Hysteroscopy	Mixed clear cell carcinoma 65%, Serous carcinoma 30% and Endometrioid carcinoma 5%	IVA	>1/2	N	Sigmoid colon
304	52	D&C	Endometrioid carcinoma FIGO G1	IA	>1/2	N	None
360	60	D&C	Endometrioid carcinoma FIGO G3	IVB	>1/2	N	Cervix, Parametrium, Omental, and bilateral accessary
429	54	Hysteroscopy	Endometrioid carcinoma FIGO G2	IA	>1/2	Y	None

### Prognosis

**FIGURE 2:**
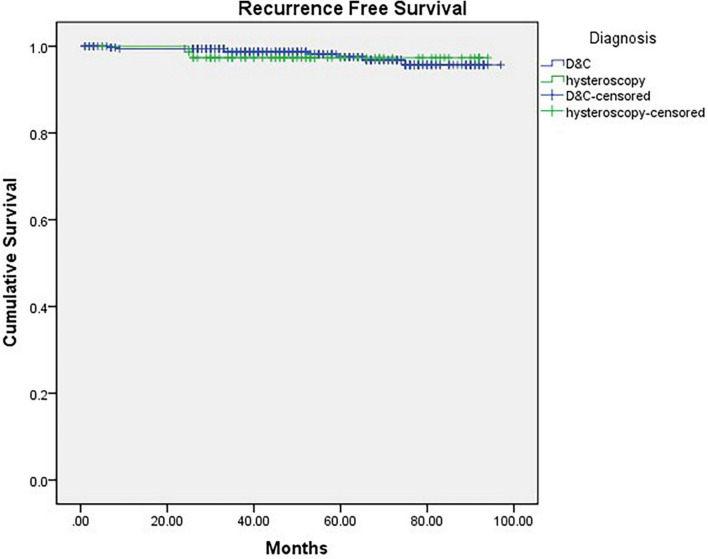
Kaplan-Meier curve of recurrence-free survival grouped by diagnostic procedure (*p* = 0.787).

**FIGURE 3:**
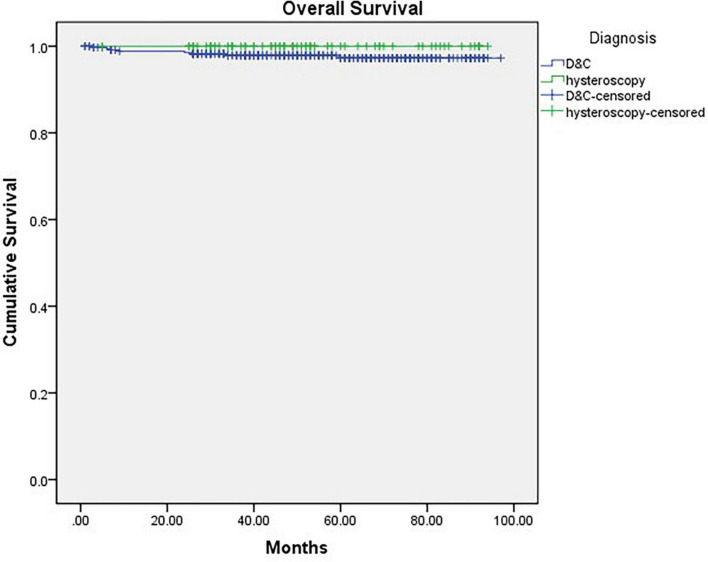
Kaplan-Meier curve of overall survival grouped by diagnostic procedure (*P* = 0.189).

Finally, we compared the OS and RFS of the two groups, and the mean follow-up time (months) of Group A was 53.605 months (CI: 48.843–58.367) and that of Group B was 58.158 months (CI: 55.956–60.367). There was no significant difference between the 2 groups (P_*os*_ = 0.189, P_*RFS*_ = 0.787) (Figures 2, 3).

## Discussion

Our findings affirm that HSC as a diagnostic procedure is not associated with a worse pathological risk factors and prognosis, and may probably be safely used in patients with endometrial cancer. Therefore, HSC may be used as one of the standard procedures for the diagnosis of suspected endometrial cancer. Additionally, HSC is considered the gold standard for evaluating the uterine cavity in cases of abnormal uterine bleeding (20), especially increased the accuracy in the diagnosis of endometrial cancer.

Up to now, the safety of HSC used in endometrial cancer, especially its long-term prognosis, are controversial. One of the most controversial points is whether hysteroscopy worsens the stage and prognosis of endometrial cancer. Therefore, this study was conducted to investigate this point. According to the hypothesis, the main cause of the worse progression of endometrial cancer may be the hysteroscopic distension pressure, which includes two key factors, inflation pressure and distension medium (21), for the spread of cancer foci into the abdominal cavity through the distension medium or for the invasion of cancer foci to a deeper level. In the present study, the inflation pressure was 20-23 kPa, and the distension medium was saline solution. The final study found no significant difference in postoperative peritoneal cytology, pathological staging (including the depth of myometrial invasion and lymph node metastasis) or prognosis of endometrial cancer detected by hysteroscopy compared with conventional curettage. It is reasonable to assume that hysteroscopic exploration and biopsy are safe, at least at the inflation pressure and distension medium used in this study. Studies in earlier years showed that hysteroscopy was associated with poor prognosis (22, 23), but in recent years studies have been more consistent with our findings whose hysteroscopic procedures with similarly inflation pressure and inflation media to ours (6, 7, 24, 25). Therefore, we consider that more standardized hysteroscopic practice was responsible for this change, as surgical techniques and equipment continue to improve. It is worth noting that general anesthesia was required for hysteroscopy in this study. In fact, this practice is also very common throughout China. On the one hand, anesthesia is relatively inexpensive in China, and on the other hand, human resources are limited to take a large number of patients to submitted to moderate parenteral sedation and a paracervical block (26). However, compared with the awake state, the patient’s operative experience is more comfortable and the surgical cooperation is higher under general anesthesia, which facilitates better observation of the entire uterine cavity and cervical canal for lesion sites and removal of biopsies. There are studies to mention the office HSC was no differ from hospital HSC as for the prognosis of EC, but it is no system review or RCTs on it (11, 27).

However, some studies have also found a positive relationship between the time interval and positive ascites rate after including the length of time between hysteroscopy and the full staging procedure, which may be due to the time required for ectopic colonization and escape immunization of disseminated tumor cells (15, 19). In the present study, the gap of hysteroscopy and surgery were mostly between 1 to 3 weeks as well as D&C. And it was finally confirmed that hysteroscopy at this interval does not worsen the prognosis by our long-term follow-up.

It is worth mentioning that whether positive peritoneal cytology worsens the prognosis of the cancer is also a point of controversy. Though several studies have shown that positive peritoneal cytology has an impact on prognosis (17, 28, 29), the other studies have shown no significant correlation (30, 31), and then in the 2009 FIGO classification system, positive peritoneal cytology is not included in the grading criteria. The NCCN guidelines also do not use peritoneal cytology as an risk-factor of prognosis and adjustment of treatment options for endometrial cancer (32). However, up to now NCCN, FIGO, and AJCC still recommend to keep the step of retention of ascites or peritoneal irrigation fluid in full staging procedures of EC, on the one hand due to its still controversial prognostic impact. On the other hand, as the main way by which hysteroscopy may promote the possibility of tumor progression. And we think the retention of ascites or abdominal irrigation fluid is also a good way to assess and exclude this possibility, especially for specific types of endometrial cancer. In an era of sentinel node and molecular classification, tumor cells which enter the pelvis through the fallopian tubes is a potential risk factor to cause disseminated metastases in the pelvic and abdominal cavities. Moreover, hysteroscopy may also influence the lymphovascular space invasion and the depth of myometrial invasion, which are prognostic risk factor of EC. Therefore, hysteroscopy is a complementary tool to sentinel node and molecular classification to further refine diagnostic staging and guide treatment.

There are also near-term adverse effects of hysteroscopy, including water toxicity, uterine perforation, adjacent organ damage, bleeding, infection and air embolism, and long-term adverse effects of uterine adhesions (33). For near-term adverse reactions, our study found that only one patient had fever with vaginal bleeding 1 week after hysteroscopy, was considered to have a uterine infection and was discharged after 1 week of anti-infection treatment.

Finally, we found that in this study, there were 58 women of reproductive age (< 45 years), 13 of whom underwent hysteroscopy, which also included two stage IIIC patients. By the end of follow-up, none of these 58 women had experienced death or recurrence. Although they all underwent full staging procedure, it provides evidence that hysteroscopy does not increase the risk of distant metastases of endometrial cancer and provides a basis for future studies of conservation therapy. This finding is consistent with current guidelines and other relevant studies recommending the use of hysteroscopic treatment for patients with early-stage endometrial cancer (3, 10).

As the largest referral hospital in Fujian Province, China, our multicenter data reflect the situation of hysteroscopic exploration and lesion biopsy for the population with endometrial cancer on the southeast coast of China. Moreover, the follow-up period of this study was up to 3 years, which can also reflect the true prognosis to some degree. Of course, there are some shortcomings in this study. For example, the sample size was still insufficient after screening. The association between duration of hysteroscopy, the time interval from hysteroscopy to full surgery and positive ascites was not further investigated due to ambiguous case data or follow-up. A retrospective case-control study showed that for patients with early endometrial cancer (FIGO I-II stage) the time between hysteroscopy and staging surgery was not statistically different between the positive and negative cytology groups (34). In the current study, we follow-up for 4 years, and no prognostic differences between the hysteroscopy and the controls. And our results showed no difference between endometrial and serous cancer.

As an obstetrics and gynecology referral hospital, we have been committed to bringing more accurate, convenient and comfortable treatment services to the local population. When we learned that pipelle sampling device, which can take with less training and less use of resources, has been shown to be as accurate as hysteroscopy (35, 36). Therefore, we are actively involved in a national clinical trial to facilitate the implementation of pipelle in China, particularly in a public health system that needs referral. Even though the trial is currently in its infancy and has a limited sample size and was not included in this study. However, we also look forward to exploring more new techniques to serve the public based on this study.

## Conclusion

Under a certain inflation pressure between 20 and 23 kPa and distension medium like saline solution, hysteroscopic exploration and lesion biopsy, as a screening test for endometrial cancer, ensure safety and have no adverse effects on prognosis compared to conventional curettage.

## Data availability statement

The raw data supporting the conclusions of this article will be made available by the authors, without undue reservation.

## Ethics statement

This study was approved by our Institution’s Ethics Committee (2022KYLLR0343). The patients/participants provided their written informed consent to participate in this study. Written informed consent was obtained from the individual(s) for the publication of any potentially identifiable images or data included in this article.

## Author contributions

HY and XZ conceived the study. SL, YC, SZ, XHL, QY, and HL were responsible for clinical data collection. SL and HY analyzed the data. SL, XZ, and HY composed the first draft of the manuscript and edited it. LZ helped SZ complete the first and second revision of this article, including add reference, shape the manuscript, and so on during the most part of author went to the front line to participate in the fight against the epidemic in China. All authors approved the final manuscript.
